# Evaluating the Impact of a Cream Containing Horse Placental Extract on Eye Corner Wrinkles in Healthy Women: Single-Blind Comparative Study

**DOI:** 10.2196/51070

**Published:** 2023-11-15

**Authors:** Tsuyuko Watanabe, Kentarou Tahara, Eiichi Hirano

**Affiliations:** 1 Medical Affairs Department Japan Bio Products Co, Ltd Tokyo Japan

**Keywords:** wrinkle, horse placental extract, corner of the eyes, eye corner, wrinkle grade standards, skin replica analysis, crow's feet, laugh lines, lateral canthal lines, canthal line, dermis, epidermis, nasolabial fold, cheek, forehead, under eye, dynamic wrinkle, static wrinkle, wrinkle fold, dermatology, dermatologist, skin, women, skin care, effective, cream, face, design, effect, eye, optician, ophthalmologist

## Abstract

**Background:**

Placental extract has been mostly used in skin care for cosmetic purposes. However, the use of various placental extracts has been limited due to the lack of established and effective application methods.

**Objective:**

In this study, we investigated the antiwrinkle effect of a cream formulation—LNC wrinkle eye cream (LNC-EC)—containing horse placental extract as the main ingredient.

**Methods:**

A total of 24 healthy women, aged 37-54 years, with wrinkle grades 1-3, were treated with LNC-EC for 2 weeks. The cream was applied on one-half of the participants’ faces, and the results were compared with the untreated half of the face.

**Results:**

Visual inspection, using the wrinkle grade standard, showed that the area treated with LNC-EC had a significantly lower wrinkle grade than the untreated area when comparing before and after the application of LNC-EC. In addition, replica analysis showed a significant reduction in both the maximum wrinkle width and the number of wrinkles in the LNC-EC–treated area in comparison to the untreated area before and after the application. These results suggest that LNC-EC has an antiwrinkle effect on the corners of the eyes based on parameters like the maximum wrinkle width and the number of wrinkles.

**Conclusions:**

LNC-EC, with horse placental extract as its main ingredient, was shown to be effective in improving wrinkles at the eye corners, presumably due to a reduction in the maximum wrinkle width and the number of wrinkles. Interpretation of the results is limited because this study was conducted only in the intervention group. A randomized controlled trial with a placebo control group is necessary to verify the antiwrinkle effects of horse placental extract.

## Introduction

Skin aging is influenced by both internal (changes in hormonal status and local immune system [1]) and external (ultraviolet exposure and effects of the regulation of the hypothalamic-pituitary-adrenal axis [1,2]) factors. The human face is constantly exposed to the outside world, influencing personal impressions [3]. Various age-related facial changes are noticeable [4], particularly periorbital area changes [5]. The skin around the orbits is thinner compared to other parts of the face [6]; wrinkles tend to form at eye corners because of frequent movements, such as blinking and facial expressions [7].

Dermatologically, placental extracts have been reported to enhance epidermal protection by promoting keratinocyte proliferation, increasing antibacterial peptide expression (eg, defensins), reducing cell damage from fine particulate matter, supporting the growth of epidermal indigenous bacteria, and inhibiting cellular aging during 5-bromo-2’-deoxyuridine induction [8,9]. Although the effectiveness of placental extracts in skin care, especially in improving wrinkles, has been reported [10,11], their practical remains undetermined due to variations in extraction and administration methods, including dosing and application, across different studies. To assess the efficacy of placental extracts, we investigated their impact on eye-corner wrinkles in healthy participants during the drier winter months.

## Methods

### Materials and Clinical Study

The LNC wrinkle eye cream (LNC-EC; Japan Bio Products Co) was used.

This randomized, single-blind clinical trial was conducted from January 20 to February 3, 2022, in Kirei Testing Labo Co, Ltd. We established a single group to compare the untreated and treated sites in the same participant. Randomization was performed to reduce bias.

### Ethics Approval

This study was reviewed and approved by the Kirei Testing Laboratory Ethics Committee on January 18, 2022 (ID 22000134) and registered in the University Hospital Medical Information Network Clinical Trials Registry (UMIN000051646). The participants signed an informed consent, which included the study purpose, methods, confidentiality, and the right to withdraw. Confidentiality was ensured. Study procedures were conducted according to the World Medical Association’s Helsinki Declaration and its amendments.

### Participants and Setting

The inclusion criteria were as follows: healthy Japanese women aged ≥20 and <59 years at the time of consent, women with wrinkle grades 1 to 3 at both eye corners, and women with skin classified as Fitzpatrick type III or IV. Exclusion criteria included the following:

- Women with markedly different wrinkle grade scores (specifically, a difference ≥ +/–1) between the left and right eye corners
- Women whose skin at the evaluation site had inflammation, trauma, contusion, pimples, warts, blemishes, or traces of diseases like atopic dermatitis or urticaria that may affect study results
- Women continuously using skin care products, cosmetics, quasi-drugs, or health foods claiming or emphasizing similar efficacy as the test product (reduction of fine lines and wrinkles caused by dryness) at the evaluated area
- Women who had undergone or planned to undergo cosmetic procedures on the evaluation site or elsewhere during the study period
- Women who changed their skin care products or started using new basic cosmetic or sunscreen products on the evaluation site within the past 4 weeks
- Women exposed to excessive ultraviolet radiation (eg, prolonged outdoor work, exercise, swimming, or leisure activities) within the past 4 weeks or planning such activities during the study
- Women participating in another study at enrollment or during the planned study duration
- Women receiving hormone replacement or any other treatments for various medical conditions at enrollment or during the study
- Women currently undergoing dermatological treatment
- Women with a history of serious diseases involving glucose and lipid metabolism; liver and renal function; cardiac, cardiovascular, respiratory, endocrine, immune, and neurological systems; or psychiatric disorders
- Women working night or a combination of day and night shifts
- Women with a history of alcohol and drug dependence
- Women who take alcohol, vitamin B_12_, or melatonin for sleeping
- Women at risk of developing allergies to cosmetics and food or who have developed skin rashes or other skin problems due to cosmetics within the past year
- Pregnant or lactating women at the time of consent or those planning to become pregnant during the study
- Women using mosaics, eye tapes, or other double-eyelid-shaping products that cannot be removed for testing
- Women who had eyelash extensions and could not tolerate the solvent used during the replica collection
- Participants deemed ineligible for any reason by the supervising physician or investigator

If any information rendering them ineligible was identified during the study, they were excluded from the analysis. A total of 24 participants were selected for the study through visual assessment based on the wrinkle grade standard (8 grades: 0-7). Informed consent was obtained from all participants. Furthermore, a consultation service was established at a medical institution to address any health-related enquiries.

### Test Product Application

Test products were randomly assigned to participants using the half-face method on symmetrical areas of the face, with no apparent bias in the left-right distribution [11]. Participants were instructed to apply the test product (0.5 g) to one-half of the face both in the morning and at night, consistently at the same time, and to continue applying the product until the final measurement date.

### Wrinkle Measurement Using Wrinkle Grade Standards

Photographic and visual assessments were conducted in accordance with the guidelines for evaluating antiwrinkle products, as authorized by the Japanese Cosmetic Science Society. Trained experts, proficient in wrinkle evaluation, assigned participants an 8-point wrinkle grade score using the wrinkle grade evaluation criteria and photographs provided by the Japanese Society of Cosmetic Science and Technology [12].

### Wrinkle Measurement Using Skin Replica Analysis

Skin replicas of the target site (10 × 10 mm or larger) were made and analyzed using a replica analysis system (ASA-03RXD; ASH, Japan) to calculate the wrinkle area and volume ratios, maximum wrinkle depth and width, average wrinkle depth, and the number of wrinkles [13].

### Statistical Analysis

The untreated and treated sides were compared by calculating the change (Δ) at 2 weeks (baseline scores minus 2-week scores, reported as the mean change from baseline for a parameter). Skin replica analysis was performed using a statistical software program built into the replica analysis system (ASA-03RXD; Nihon Ash Co, Ltd). For each clinical parameter, the groups were compared using the Wilcoxon signed rank test and a paired 2-tailed *t* test (*P*=.05) for wrinkle and replica analysis, respectively, to determine statistically significant differences in improvement between the untreated and treated sides.

## Results

### LNC-EC Effects on Skin Wrinkles According to Wrinkle Grade Standards

**Figure 1:**
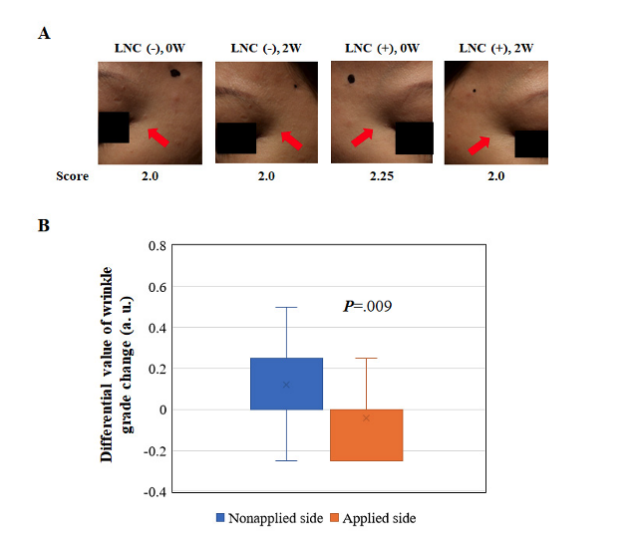
Assessment based on the wrinkle grading standards. (A) Representative example comparing the untreated and treated sides with LNC wrinkle eye cream before (baseline) and after 2 weeks of treatment. Photographs illustrate the improvement in facial wrinkles. Arrows indicate the specific areas of interest. (B) Box plot representation of the differential value of wrinkle grade change (arbitrary unit: a. u.) between the untreated and treated sides with LNC eye cream before (baseline) and after 2 weeks of treatment.

The wrinkle grade on the treated side was 2.25 at baseline but decreased to 2.0 after 2 weeks of use, resulting in less noticeable wrinkles. On the untreated side, the grade remained at 2.0 from baseline to the end of the 2-week period (Figure 1A). A significant difference was observed when subtracting the pretest from the posttest assessment value based on the wrinkle grade standard (Figure 1B).

### LNC-EC Effects on Skin Wrinkles Based on Skin Replica Analysis

**Figure 2:**
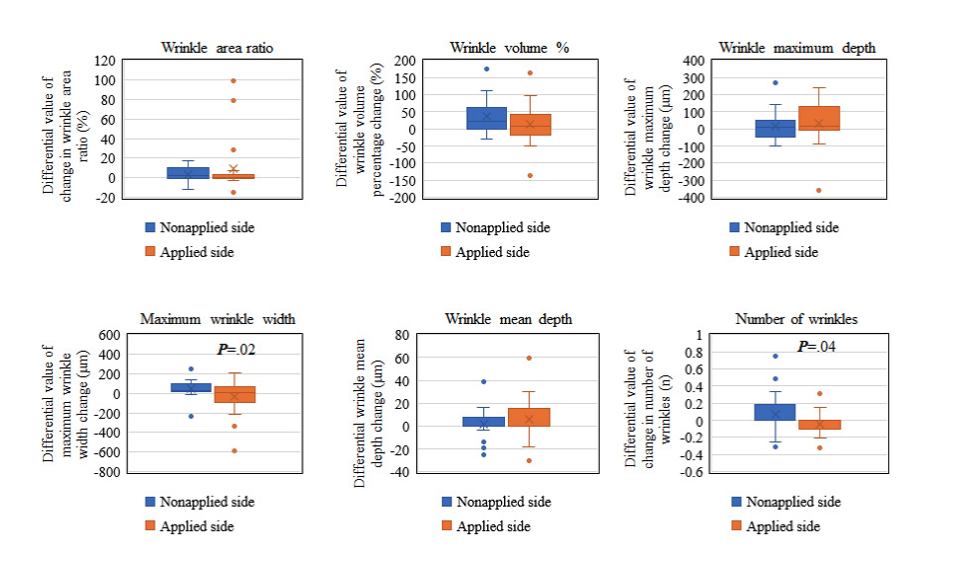
Box plot representation of the differential value of various parameters between the nonapplied and applied sides of the skin when using LNC wrinkle eye cream, both before (0 weeks) and after 2 weeks of treatment.

No significant differences were observed in pre- and posttest results for wrinkle area ratio, wrinkle volume percentage, maximum wrinkle depth, and wrinkle mean depth. Meanwhile, a significant difference was detected in pre- and posttest results for maximum wrinkle width and the number of wrinkles (Figure 2).

## Discussion

### Principal Findings

In this study, LNC-EC was applied to healthy participants for 2 weeks to verify its cosmetic effects. Both wrinkle grade evaluation and replica analysis demonstrated its antiwrinkle effects at the eye corners. The replica analysis revealed a reduction in the maximum width and the number of wrinkles. These results suggest that horse placental extract has cosmetic benefits.

Wrinkles can result from external factors like dryness due to decreased temperature and humidity as well as internal factors related to reduced moisture retention due to aging. This study was conducted during winter when temperature and humidity levels are at their lowest. Consequently, external factors could potentially compromise the barrier function of each skin layer. The stratum corneum’s barrier function weakens during winter due to smaller corneocytes compared to summer [14]. Epidermal dryness leads to structural changes causing shallow wrinkles, so preventing dryness is crucial in improving wrinkles. Horse placental extract enhances skin barrier function by promoting the growth of *Staphylococcus epidermidis* and antimicrobial peptide expression in human epidermal keratinocytes [8]. Furthermore, aged human keratinocytes exhibit increased stratifin expression, suppressing filaggrin and serine palmitoyltransferase involved in epidermal moisturization [15]. Horse placenta extract reduces stratifin expression in aged human cultured epidermal cells [9], suggesting that strengthening the skin barrier with this extract may help prevent worsening wrinkles.

Ultraviolet B stimulates epidermal keratinocytes, resulting in the release of inflammatory cytokines (interleukin [IL]-1α, IL-6, and tumor necrosis factor-α), which stimulate dermal fibroblasts and keratinocytes to increase matrix metalloproteinase (MMP)-1, MMP-3, and MMP-9—enzymes that degrade collagen and elastic fibers, causing wrinkles [16]. Horse placenta extract suppresses IL-6 expression, which increases with age, in cultured aged human epidermal cells [9]. This suggests that the extract may indirectly mitigate the worsening of wrinkles by preventing increased MMP expression. Considering these factors, LNC-EC’s antiwrinkle effects in this study likely result from the placental extract’s various biological activities.

### Conclusions

LNC-EC appears effective in improving the appearance of eye-corner wrinkles, presumably by reducing their maximum width and number. No adverse events associated with LNC-EC application were observed during the study, indicating its safety for use.

## Abbreviations

IL: interleukin
LNC-EC: LNC wrinkle eye cream
MMP: matrix metalloproteinase
